# New Insights on Primary and Secondary Metabolite Contents of Seven Italian Wild Food Plants with Medicinal Applications: A Comparative Study

**DOI:** 10.3390/plants12183180

**Published:** 2023-09-05

**Authors:** Stefania Monari, Maura Ferri, Mirko Salinitro, Annalisa Tassoni

**Affiliations:** Department of Biological, Geological and Environmental Sciences, University of Bologna, Via Irnerio n. 42, 40126 Bologna, Italy; stefania.monari2@unibo.it (S.M.); maura.ferri@unibo.it (M.F.); mirko.salinitro2@unibo.it (M.S.)

**Keywords:** antioxidant activity, medicinal plants, polyphenols, wild food plants

## Abstract

Wild food plants are widely consumed all over the world and many have both nutritional and therapeutic value due to the presence of biologically active compounds. The present research, for the first time, aims to compare primary and secondary metabolite levels among different plant organs (flower, leaf, stem, root, bark) of seven species (*Borago officinalis* L., *Cynodon dactylon* (L.) Pers., *Foeniculum vulgare* Mill., *Hypericum perforatum* L., *Malva sylvestris* L., *Sambucus nigra* L., *Urtica dioica* L.) collected in three different Italian regions (Liguria, Tuscany, Apulia). Plant organ samples were extracted with water or 95% (*v*/*v*) methanol and liquid fractions were analyzed using spectrophotometric assays. The best results were obtained for *Hypericum perforatum* L. samples, followed by *Sambucus nigra* L. and *Borago officinalis* L. As also confirmed via PCA analysis on normalized data, flower and leaf extracts of all species exhibited higher levels of polyphenols (up to 105.7 mg GA eq/gDW), reducing sugars (up to 389.2 mg GLUC eq/gDW), proteins (up to 675.7 mg BSA eq/gDW) and of antioxidant capacity (up to 263.5 mg AA eq/gDW). No differences among the regions of gathering were detected after spectrophotometric assays, which was confirmed via PCA analysis. These data contribute to further validate the traditionally reported healing effects of these species on human health.

## 1. Introduction

Wild food plants are native species which grow spontaneously in the environment without being cultivated [1]. These edible species have been widely consumed all over the world and constitute a substantial part of the human diet [2]. In the past, their importance increased during famine periods, but after industrialization from 1950 onward, this kind of traditional knowledge has been progressively disappearing [3]. In the last few decades, these plant species have been revalued and are receiving considerable increasing attention in general by ethnobotanists and nutritionists [4,5,6].

Nowadays, wild plants are used in many food recipes, feed, production of drugs (for human and veterinary medicine), household items and ritual uses [7]. Focusing on human health applications, several ethnobotanical papers have recorded the worldwide use of spontaneous edible plants for medicinal purposes [6,8]. Due to the presence of several biologically active compounds [9], these species have been reported to have both nutritional and therapeutic value, and are therefore being considered as food–medicines. Plants are in fact rich in a wide variety of phytochemicals [10], and herbal preparations for medicinal usage contain various types of primary and secondary metabolites, such as, among others, sugars, phenolic acids, flavonoids, tannins, proteins [11]. Among secondary metabolites, polyphenols have been proven to have biological activities in humans like antioxidant, anti-inflammatory and antimicrobial capacities [12,13,14], and properties against chronic or degenerative diseases [15], while primary metabolites, such as sugars and proteins, show a considerable nutritional value [16]. Unrefined sugars show higher nutritional quality and antioxidant capacity with respect to refined ones and are composed in large amounts of reducing sugars (e.g., glucose and fructose) [17] that have beneficial effects, especially when combined with secondary metabolites such as polyphenols and flavonoids.

The synthesis and accumulation of different plant metabolites not only depends on the species but also on the environmental conditions in which the plant itself is growing. External factors such as light, temperature, moisture, and soil characteristics can affect the phytochemical profile of a plant organism [18]. A comprehensive plant metabolomic analysis needs to consider the habitat, and a comparison of the effects of different environmental factors is mandatory. Over the last few years, several phytochemical studies were published on Italian wild edible plants with therapeutic uses [6]. These studies reported data on specific plants in a restricted sampling area [19], on two or more species gathered in various locations of the same region [20], or cultivated plants [21]. To the best of our knowledge, the present paper is the first comparing different organs of diverse wild edible/medicinal plants across different Italian regions.

Recently, the Italian ethnobotanical studies published in international journals between 1980 and March 2022 and dealing with wild and cultivated food plants known to have therapeutical properties were reviewed [6]. The results highlighted the presence of 13 wild and/or cultivated plants widespread all over the Italian peninsula and showing a high number of citations and of different medicinal uses. Among them, seven wild plant species (*Borago officinalis* L., *Cynodon dactylon* (L.) Pers., *Foeniculum vulgare* Mill., *Hypericum perforatum* L., *Malva sylvestris* L., *Sambucus nigra* L., *Urtica dioica* L.), were selected for the present comparative study given the fact that they were all spontaneously growing wild and present in the similar sampling areas all along the Italian peninsula. For all of them, both food and therapeutic characteristics have been previously reviewed [1,6,22].

*B. officinalis* is traditionally used as a food in salads, in soup (alone or with beans or with mixed vegetables), in pancakes and in pies [3,23]. However, it was also widely reported in ethnobotanical research for its diuretic, emollient, expectorant, diaphoretic, anti-inflammatory and liver wellbeing properties, and its properties against several digestive system diseases, mainly via ingestion of raw and boiled leaves and cooking water, or by topical use [6,23].

*C. dactylon* is used as substitute for coffee [24,25] or tea [26], and its raw roots are eaten in salad [22]. Roots and rhizomes are also used in decoction form for a wide variety of diseases (e.g., cystitis, hepatitis, renal stones, cough, flu, fever, rheumatisms, dysmenorrhea, abscesses, prostatitis), and the whole plant extract is used both orally and topically against respiratory disorders and skin diseases [6].

*F. vulgare* is widely consumed in cooking and largely used in folk medicine. It is eaten raw or cooked, alone or in mixed vegetable salad or soups, and is commonly used as flavoring (e.g., seeds to aromatize salami giving them a recognizable taste), and in liqueur production [3,23]. Fennel is also used for gastrointestinal disorders, respiratory issues, female ailments and as an anti-pyretic, antirheumatic and detoxifier [6,23].

*H. perforatum* finds culinary applications mainly in liqueur production (i.e., flowers for grappa flavoring) or as herbal tea and food supplement [22,27,28]. Many more applications are found for *Hypericum* as a medicinal plant. Its flowers are widely used in phytotherapy all over the world for a wide range of diseases, as an infusion (e.g., to aid digestion, as an anti-depressant, to treat constipation and sleep disorders), macerated in olive oil (e.g., applied to burns, sores, skin rash, wounds, contusions, and erythema) or as decoction (e.g., against gastrointestinal and hepatic colic and as a digestive and blood depurative) [6]. Leaves and other aerial parts are used for menstrual pains, arthrosis, joint pain and rheumatisms [6].

*M. sylvestris* is used in several traditional pasta food recipes [22,29], eaten with other boiled vegetables in a salad or in a soup [4], or utilized as a herbal tea [30]. Mallow is also reported to have many different therapeutical uses. Flower and leaf decoctions and infusions are useful for skin and mouth inflammations, gastrointestinal diseases, vaginal and urogenital system inflammations and respiratory ailments, while root decoction is a remedy for cough, sore throat, stomach ache, toothache, menstrual pain, hypertension, dermatitis and weakness [6].

*S. nigra* flowers and, to a lesser extent, fruits are diffused in omelet, pancake, jam and liqueur recipes [3,23]. Sometimes, the same food preparations are used as folk medicine, such as jam reported as an antirheumatic remedy in the North of Italy [3]. Moreover, several different preparations (decoction, infusion, syrup, cataplasm) made with *S. nigra* flowers and bark are used against a wide range of respiratory, digestive, metabolic affections and skin diseases [6].

*U. dioica* is one of the most consumed wild species, both as food and medicine, being much more valued today than in the past. As food, it is eaten in mixed vegetable soups and salads, omelets, is used to make green pasta or to fill and season hand-made pasta [3,23]. As medicine, nettle is used both internally after ingestion (e.g., as expectorant, diuretic, depurative, digestive and to treat anemia, cold and coughs) and externally topically (e.g., to treat dandruff and hair loss, dermatitis, painful joints, rheumatisms, chilblains, wounds) [6].

In the present research, the seven targeted wild plant species were collected in three different Italian regions (Liguria, Tuscany and Apulia) and selected organs were characterized for their primary and secondary metabolite levels and for antioxidant capacity. The main objectives of the present research were (i) to perform a preliminary biochemical screening by quantifying total polyphenols, reducing sugars and proteins and antioxidant activity, and to validate results by means of PCA analysis; (ii) to investigate the variability of the targeted compounds into the different plant organs traditionally consumed and/or used as medicinal remedies; (iii) to assess, within the same plant species and organs, possible differences linked to the gathering regions.

## 2. Results and Discussion

**Figure 1:**
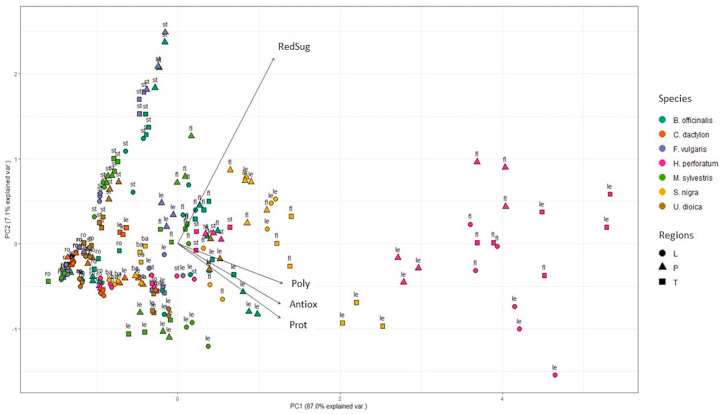
PCA analysis of spectrophotometric results on water extracts, showing the grouping of species according to total polyphenols, reducing sugars, proteins and antioxidant activity data (Figure 2). L: Liguria; P: Apulia; T: Tuscany; fl: flowers; le: leaves; st: stems; ro: roots; ba: bark; RedSug: reducing sugars; Poly: polyphenols; Antiox: antioxidant activity; Prot: proteins.

**Figure 2:**
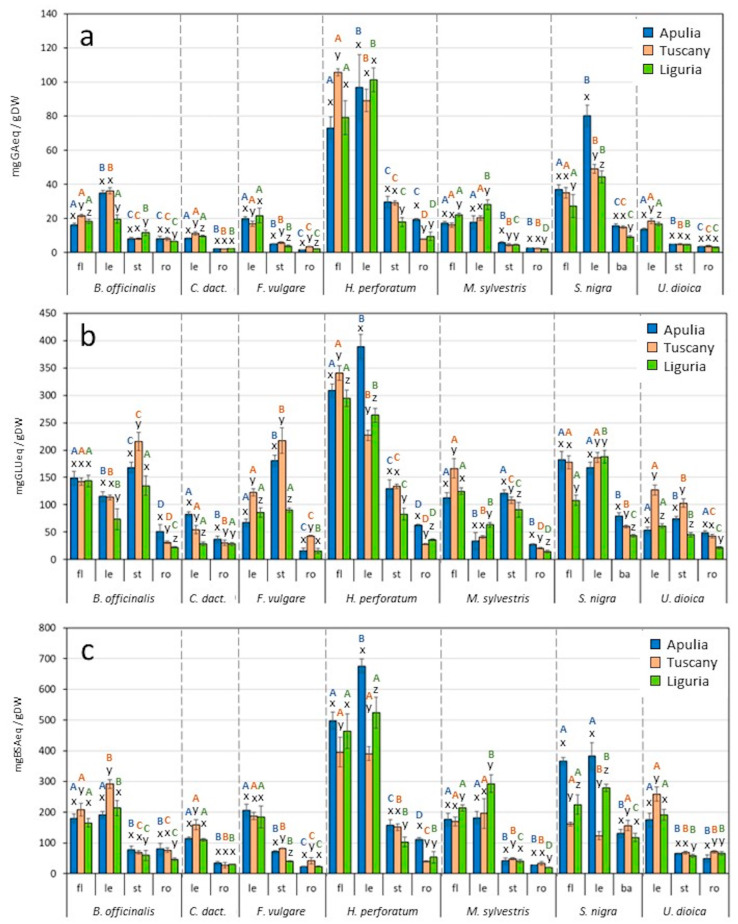

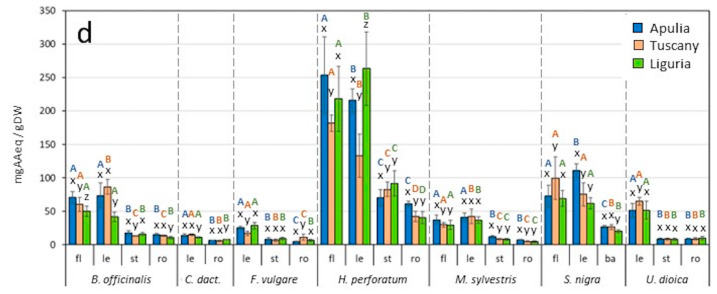
Total amounts of polyphenols (**a**), reducing sugars (**b**), proteins (**c**) and antioxidant activity (**d**) of aqueous extracts after spectrophotometric analysis. fl: flowers; le: leaves; st: stems; ro: roots; ba: bark; GA: gallic acid, GLU: D-glucose, BSA: bovine serum albumin, AA: ascorbic acid, eq: equivalent, DW: dry weight. Different letters indicate a statistically significant difference (one-way ANOVA followed by post hoc Tukey HSD test or Kruskal–Wallis followed by Dunn test, *p* < 0.05) among the regions (x, y, z) and among the organs of each species (A, B, C, D). Data are the mean ± SD (n = 3).

The biochemical characterization of the different organs of the selected species was performed with specific spectrophotometric assays (Figure 1 and Figure 2). Since these wild food plants are mainly used as herbal teas (like infusion or decoction) for medicinal purposes [6], the analyzed aqueous extracts here represent the samples closer to the actual way of usage. Additionally, methanol extracts were obtained and analyzed (Supplementary Materials, Figures S1–S3, Table S1). This paper reports for the first time the levels of several bioactive compound classes in different organs of seven wild edible/medicinal plants collected across three different Italian regions.

All data on water extracts after metabolite spectrophotometrical analysis were elaborated with Principal Component Analysis (PCA) (Figure 1) demonstrating that *H. perforatum* and *S. nigra* were the most active species and had the best total polyphenols, proteins and antioxidant activity results, while *F. vulgare* and *B. officinalis* stems showed the highest reducing sugar values.

The same PCA approach was adopted on methanolic sample data (Figure S1) and similar result clusters were highlighted. No differences among samples of the three regions were highlighted.

### 2.1. Total Amount of Polyphenols

**Figure 3:**
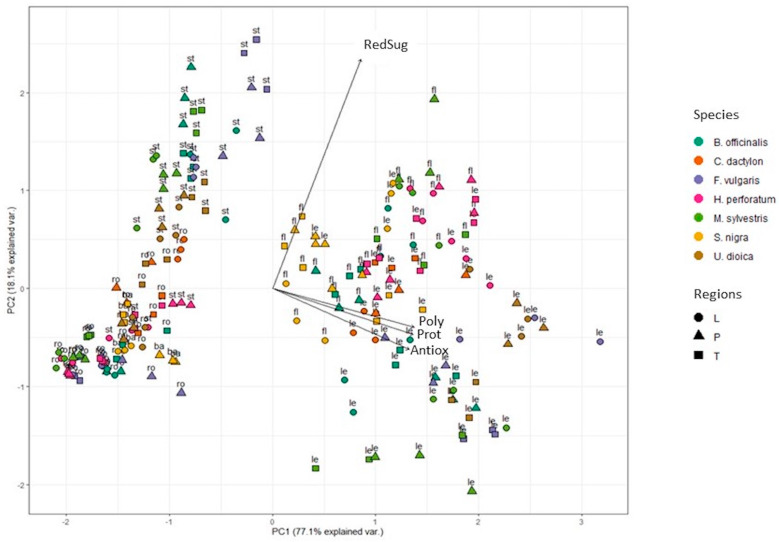
PCA analysis of aqueous extract spectrophotometric results (Table S1) after normalization (see Section 3.5) showing the grouping of organs according to the concentrations of reducing sugars, polyphenols, proteins and antioxidant activity. L: Liguria; P: Apulia; T: Tuscany; fl: flowers; le: leaves; st: stems; ro: roots; ba: bark; RedSug: reducing sugars; Poly: polyphenols; Antiox: antioxidant activity; Prot: proteins.

The total amount of polyphenols was found to be significantly higher in aqueous extracts of flowers and leaves of all analyzed plant species with respect to stems, roots and bark (Figure 2a). Similar data were observed in methanolic extracts (Figure S2a). This trend was clearly pointed out via PCA analysis on normalized values (Table S1), which were used to smooth differences among species and to highlight those among organs (Figure 3 and Figure S3). After data normalization, in fact, flowers and leaves of all the plant species were clustered together at positive PC1 values.

Samples were similarly grouped when considering aqueous (Figure 3) and methanolic (Figure S3) extracts. No general differences could be clearly evidenced among similar samples of the three gathering regions.

Overall, aqueous extracts of *H. perforatum* L. showed the highest total polyphenol content with an average of 85.9 mg GA eq/gDW in flowers and of 95.9 mg GA eq/gDW in leaves. The data were not significantly different among regions (Figure 1) except for flowers from Tuscany (105.7 mg GA eq/gDW, *p* < 0.01) (Figure 2a). This species is extensively used as medicinal plant and is already known to have high polyphenol contents [31]. Reported results were in agreement with those of Sekeroglu et al. [32], who observed higher polyphenol concentrations in *H. perforatum* flowers and leaves with respect to stem aqueous extracts. According to the literature, in *H. perforatum* the most abundant phenolic compounds showing biological activity are flavonoids, such as quercetin, rutin, kaempferol and naringenin, and phenolic acids, like chlorogenic, gallic, caffeic, ferulic and vanillic acids [33,34].

*S. nigra* L. leaves scored the second-best total polyphenol content, with Apulia samples (80.3 mg GA eq/gDW) showing almost double the level of Tuscany and Liguria (49.1 and 44.6 mg GA eq/gDW, respectively), and with an average 1.7-fold significantly higher content with respect to flowers (on average 33.1 mg GA eq/gDW, *p* < 0.01) (Figure 2a). The present results seem to be totally comparable to those reported by Viapiana and Wesolowski [35] that detected a maximum 35.57 mg GA eq/gDW total amount of polyphenols in water infusions of *S. nigra* flowers.

Polyphenol levels of leaves and flower water extracts of *B. officinalis* L. (on average 30.1 and 18.7 mg GA eq/gDW, respectively) were seven-fold higher than those obtained by Karimi et al. [36] on leaves of the same species. Antioxidant and anti-inflammatory activities of *S. nigra* L. have been related to the presence of anthocyanins, phenolic acids and flavonoids (such as gallic acid, rutin and quercetin) [35,37], while in *B. officinalis* L. to syringic and rosmarinic acids, myricetin, rutin and kaempferol [38,39].

The results obtained from methanolic extracts (Figure S2a) showed the same trend detected in water extracts (Figure 2a) with slightly higher extraction efficiency given to the use of methanol organic solvent, as well documented in the literature [40]. In particular, after methanol extraction, polyphenol concentrations in flowers and leaves of *H. perforatum* and *S. nigra* were three to four-fold more concentrated respect to the aerial parts of methanolic extracts of some other wild plants (e.g., *Hypericum montbretii* Spach., *H. bupleuroides* Griseb., *Apium graveolens* L. and *Coriandrum sativum* L.) [41,42].

### 2.2. Total Amount of Reducing Sugars

The content of reducing sugars in water and methanolic extracts of different plant organs was investigated (Figure 2b and Figure S2b) and data were normalized and elaborated using PCA (Figure 3 and Figure S3). After water extraction (Figure 3), samples of stems, flowers and leaves of all species were those more enriched in reducing sugars, while roots and bark had lower amounts. Analogous results were obtained in methanolic samples (Figure S3). In general, no statistically significant differences were detected among regions (Figure 3), with the exception of leaves of *H. perforatum* collected in Apulia, which showed reducing sugar levels of 389.2 mg GLU eq/gDW, respectively 1.7 and 1.5 times higher than in leaves from Tuscany and Liguria, respectively. No previous publication has reported the total amount of reducing sugars in water extracts of *H. perforatum.* Jaradat [43] quantified these compounds only in methanolic extracts of *Hypericum lanuginosum* Lam; Nahdi et al. [44] determined the reducing sugar contents in methanolic and aqueous extracts of *Hypericum humifusum* L. leaves showing a 3.7-fold higher content in the methanolic (1.9 g/L) with respect to the aqueous extract (0.51 g/L).

Among other species, *B. officinalis* and *F. vulgare* stems showed the highest reducing sugar levels (172.8 and 162.5 mg GLU eq/gDW, respectively), on average 1.7-, 1.2- and 5.7-fold higher than leaves, flowers and roots, respectively. The results from *F. vulgare* are in agreement with previous data from the literature [45], in which stems showed higher concentrations of reducing sugars (1.49 g/100 g) compared to leaves (0.72 g/100 g). Similar levels of reducing sugars were previously detected in other medicinal plants such as *Arisaema erubescens* (Wall.) Schott (134.47 mg/g DW) and *Lindera neesiana* (Wall. ex Nees) Kurz (176.72 mg/g DW) [46].

Data on methanolic extracts (Figure S2b) reported the highest concentrations of sugars in flowers and leaves of *H. perforatum* (on average 210.9 and 251.8 mg GLU eq/gDW, respectively) and in stems of *B. officinalis* and *F. vulgare* (on average 1.3 to 1.6-fold higher than flowers and leaves of the same species, respectively).

### 2.3. Total Amount of Proteins

The amount of total proteins was higher in flowers and leaves compared to other plant organs, both in aqueous and methanolic samples (Figure 2c, Figure 3, Figures S2c and S3). These results are in agreement with previous studies on different wild food and medicinal plants which detected higher levels of proteins in leaves and flowers with respect to stems and roots [47,48].

In aqueous extracts, the highest protein level was detected in *H. perforatum* flowers (from 395.5 to 497.8 mg BSA eq/gDW, respectively in Tuscany and Apulia samples) and in leaves (from 390.2 to 675.7 mg BSA eq/gDW, respectively in Tuscany and Apulia samples). The few studies that have reported the total protein amount in *H. perforatum* [49,50,51] were carried out with extracts obtained with different methods to the present paper. In particular, Karppinen et al. [51], by means of an extraction with a sodium borate buffer, obtained higher protein yields in flowers and leaves with respect to stems and roots of *H. perforatum*, in accordance with data here reported in Figure 2c.

On average, flowers and leaves of *S. nigra* from Apulia showed 2.7-fold higher protein levels than samples from Tuscany, and 1.5-fold higher than samples from Liguria (*p* < 0.01). Interestingly, the present results are comparable to those obtained by Bosi et al. [52] on different leguminous, well-known important sources of vegetable proteins. Besides the previously mentioned results, no significant difference was detected among the three regions (Figure 1).

Methanol extracts showed the highest protein yield in all *H. perforatum* organs (with maximum levels in flowers, 800.6 mg BSA eq/gDW on average) with respect to all other samples (Figure S2c).

### 2.4. Antioxidant Activity

The antioxidant activity of aqueous and methanolic extracts was evaluated (Figure 2d and Figure S2d). *H. perforatum* showed the highest activity, mainly in water samples of flowers from Apulia and Liguria (253.6 and 218.3 mg AA eq/gDW, respectively) and of leaves of the same regions (216.1 and 263.5 mg AA eq/gDW). Overall, the present results show a higher antioxidant activity in *H. perforatum* samples compared to those of well-known food plants such as *Brassica rapa* L. (0.12 mg AA eq/gDW) [53]. With the exception of the previously reported data, no differences between organs of different regions were detected (Figure 1). In agreement with present results, previous reports showed higher antioxidant activity in aqueous extracts of *H. perforatum* aerial parts respect to those of other wild plant species [54]. Previous reports also showed a higher antioxidant concentration in leaves and flowers of *S. nigra* [55,56], in line with data reported in Figure 2d, where flowers and leaves scored on average 80.5 and 82.8 mg AA eq/gDW, respectively, followed by flowers and leaves of *B. officinalis* (60.5 and 67.3 mg AA eq/gDW).

Similarly to water samples, in methanol extracts (Figures S2d and S3), the highest antioxidant activity was detected in flowers, leaves and stems of *H. perforatum* (182.3, 176.4 and 90.9 mg AA eq/gDW, respectively), followed by flowers and leaves of *S. nigra* (83.9 and 72.1 mg AA eq/gDW, respectively). After PCA analysis of aqueous (Figure 3) and methanolic (Figure S3) normalized data, leaves and flowers of all species exhibited a stronger antioxidant activity respect to stems, roots and barks, in accordance with total polyphenol concentrations (Figure 2a and Figure S2a).

## 3. Materials and Methods

### 3.1. Study Areas

**Figure 4:**
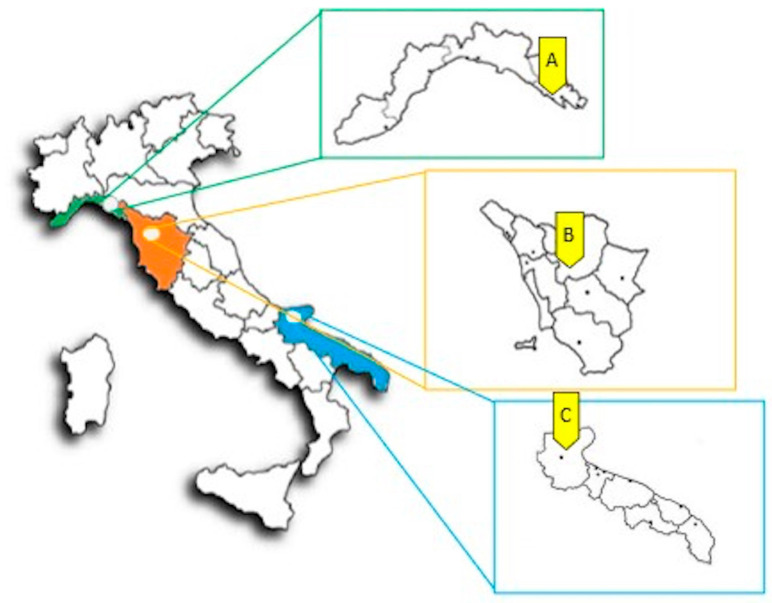
Map of the study areas. (**A**): Riomaggiore (Liguria region); (**B**): Monte Ferrato (Tuscany region); (**C**): Gargano (Apulia region).

Three Italian regions were selected on the basis of their geographical North–South and East–West positioning in the Italian peninsula: Liguria (northeast), Tuscany (East-central), Apulia (southwest). For each region, a search on Wikiplantbase #Italia website (http://bot.biologia.unipi.it/wpb, accessed on 5 March 2021) was performed in order to choose a collection area as small as possible in which all the seven species could be located together. Specifically, the areas of Riomaggiore 44°06′01.6″ N, 9°44′05.7″ E (Liguria), Monte Ferrato 43°55′23.3″ N, 11°05′04.7″ E (Tuscany) and Gargano 41°51′39.2″ N, 15°21′03.4″ E (Apulia) were selected (Figure 4).

### 3.2. Plant Material

In a previous paper, a complete database of Italian wild and/or cultivated edible plants also showing medicinal effects was created [6]. Results pointed out 13 wild and/or cultivated plant species being highly cited (citation index) and with a high number of reported medicinal uses (use index). Based on the previous findings, in the present study, only the wild species were selected and specifically: *Borago officinalis* L., *Cynodon dactylon* (L.) Pers., *Foeniculum vulgare* Mill., *Hypericum perforatum* L., *Malva sylvestris* L., *Sambucus nigra* L., *Urtica dioica* L.

**Figure 5:**
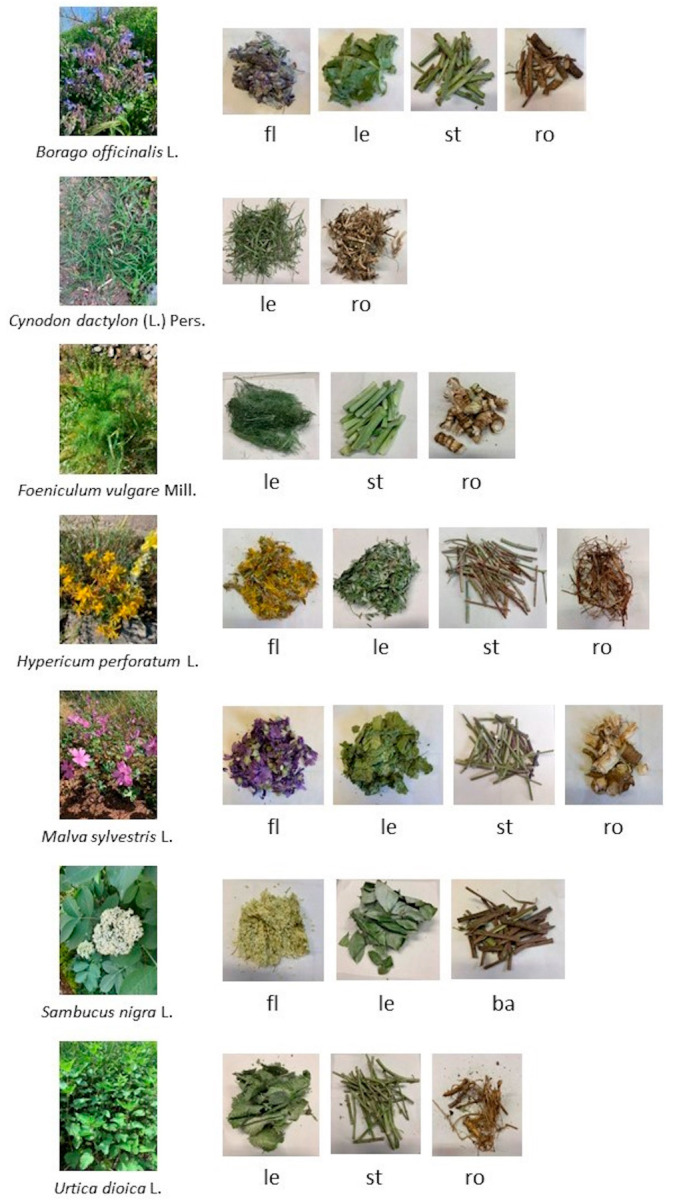
Selected species and different organs analyzed. Fl: flowers; le: leaves; st: stems; ro: roots; ba: bark (Photos by S. Monari).

Sampling was carried out, by following the geographic coordinates provided by Wikiplantbase #Italia (http://bot.biologia.unipi.it/wpb, accessed on 5 March 2021), between April and May 2021. The plants were collected in all the regions at the following phenological stages: *B. officinalis*, *H. perforatum*, *S. nigra* and *M. sylvestris* at advanced flowering; *C. dactylon* at vegetative stage with blossom starting to appear; *F. vulgaris* at vegetative phase (stem elongation 40–60 cm), *U. dioica* at vegetative phase (stem elongation 90–110 cm). Different individual plants (5–6) for each species in each sampling area were gathered and pooled together in order to have the same homogeneous starting sample for the following analysis. The collected plants were stored in water to preserve their conditions during travel back to laboratory, and immediately washed and separated into different organs (flowers, leaves, stems, roots, and when present bark, Figure 5). Each sample was frozen in liquid nitrogen, lyophilized and grinded with a blender, and the dry powder was stored at room temperature until further analysis.

### 3.3. Sample Extraction

For each organ, three biological replicates (0.2 g DW) were extracted with 100% (*v*/*v*) MilliQ water at 40 °C, 1 h shaking, with a S/L of 1:40, and with 95% (*v*/*v*) methanol at room temperature, 5 min shaking, with a solid/liquid ratio (S/L) of 1:20 [57]. The resulting extracts were then centrifuged at 5000 rpm for 10 min at 20 °C to separate the solid and liquid fractions. The latter were stored at −20 °C until spectrophotometric analyses.

### 3.4. Spectrophotometrical Analyses

Liquid aqueous and methanol extracts were analyzed by means of different spectrophotometric assays to assess the total amount of polyphenols, reducing sugars and proteins and antioxidant activity. Total polyphenol content was determined using the Folin–Ciocalteu method [58]. Results were expressed as mg of gallic acid (GA) equivalent per g of dry weight (mg GA eq/gDW) by means of a dose–response calibration curve (between 0 and 15 µg of GA). Total reducing sugar content was measured with the 3,5-dinitrosalicylic acid (DNS) method [59]. Results were expressed as mg of D-glucose (GLU) equivalent per g of dry weight (mg GLU eq/gDW) by means of a dose–response calibration curve (between 50 and 500 µg of GLU). Total protein content was determined as described by Lowry et al. [60]. Results were expressed as mg of bovine serum albumin (BSA) equivalent per g of dry weight (mg BSA eq/gDW) by means of a dose–response calibration curve (between 0 and 200 µg of BSA). Antioxidant activity was assessed using the ABTS (2,2′-azino-di-(3-ethylbenzthiazoline sulfonic acid)) method [5,32]. Results were expressed as mg of ascorbic acid (AA) equivalent per g of dry weight (mg AA eq/gDW) by means of a dose–response calibration curve (between 0 and 2 µg of AA).

### 3.5. Statistical Analyses

All assay procedures were performed on three biological replicates, each of which were analyzed in four technical replicates. The results were expressed as the mean (n = 3) ± SD per g DW (Figure 4 and Figure S2). Data were tested for normality using the Shapiro–Wilk test, and for homogeneity of variance using the Levene’s test with default parameters from the package *car* (https://CRAN.R-project.org/package=car, accessed on 3 February 2023) [61]. Differences in polyphenol, reducing sugar and protein contents, and in antioxidant activity, were evaluated among plant organs and gathering regions (within the same species). When data were parametric, one-way ANOVA followed by a Tukey HDS test (*p* < 0.05) was used, while when data were not parametric, the Kruskal–Wallis test followed by the Dunn test was applied (*p* < 0.05). Principal Component Analysis (PCA) was performed to highlight the differences among species using original values for polyphenols, reducing sugars, proteins and antioxidant activity (Figure 3 and Figure S1). Normalized values (Table S1) with respect to the average of the species in a given region were calculated as follows, and used for PCA analysis (Figure 5 and Figure S3) to highlight differences between plant organs:

$$\mathrm{Poly}\_{\mathrm{Norm}\;}_{sample\;A,sp1,r1}=\frac{0.5\;\cdot {\;\mathrm{Poly}\;}_{sample\;A,sp1,r1}}{{\mathrm{average}\;\mathrm{Poly}\;}_{sp1,r1}}$$

$$\mathrm{RedSug}\_{\mathrm{Norm}\;}_{sample\;A,sp1,r1}=\frac{0.5\;\cdot {\;\mathrm{RedSug}\;}_{sample\;A,sp1,r1}}{{\mathrm{average}\;\mathrm{RedSug}\;}_{sp1,r1}}$$

$$\mathrm{Prot}\_{\mathrm{Norm}\;}_{sample\;A,sp1,r1}=\frac{0.5\;\cdot {\;\mathrm{Prot}\;}_{sample\;A,sp1,r1}}{{\mathrm{average}\;\mathrm{Prot}\;}_{sp1,r1}}$$

$$\mathrm{Antiox}\_{\mathrm{Norm}\;}_{sample\;A,sp1,r1}=\frac{0.5\;\cdot {\;\mathrm{Antiox}\;}_{sample\;A,sp1,r1}}{{\mathrm{average}\;\mathrm{Antiox}\;}_{sp1,r1}}$$

All statistical analyses were performed using R software version 3.5.1 (R Core Team, Vienna, Austria).

## 4. Conclusions

Seven Italian wild edible plants (*Borago officinalis* L., *Cynodon dactylon* (L.) Pers., *Foeniculum vulgare* Mill., *Hypericum perforatum* L., *Malva sylvestris* L., *Sambucus nigra* L., *Urtica dioica* L.), also known for their multiple therapeutic uses, were selected and analyzed to investigate the variability of health-related compounds in the different traditionally consumed plant organs, and at assessing possible differences linked to the different gathering regions. Results indicated that (i) the highest levels of polyphenols, reducing sugars and proteins, as well as of antioxidant activity, were detected in the extracts of *H. perforatum* L. (on average up to 105.7 mg GA eq/gDW, 389.2 mg GLUC eq/gDW, 675.7 mg BSA eq/gDW and 263.5 mg AA eq/gDW, respectively), followed by *S. nigra* L. and *B. officinalis* L. extracts; (ii) flowers and leaves of all plant species exhibited a higher amount of bioactive compounds with respect to stems, roots and bark (e.g., total polyphenols of *H. perforatum* flowers and leaves from Tuscany were 13.6- and 11.5-fold higher than in roots, respectively), and these correlated with higher antioxidant potential; (iii) in general, very few differences were detected among samples collected in different regions along the Italian peninsula.

In the present paper, for the first time, seven different Italian wild edible species collected in three different regions were compared, enhancing the knowledge of some of the most widely and traditionally used food–medicine plants by providing additional evidence on the presence of bioactive compounds in their different organs. However, further research is needed to directly correlate the metabolite data with the traditionally reported health effects of these plants, and furthermore to validate them as possible substitutes of chemical drugs for therapeutic applications.

## Data Availability

All data are available withing the manuscript of Supplementary Materials.

## Supplementary Materials

The following supporting information can be downloaded at: https://www.mdpi.com/article/10.3390/plants12183180/s1, Figure S1: PCA analysis of spectrophotometric results on methanol extracts, showing the grouping of species according to total polyphenols, reducing sugars, proteins and antioxidant activity data. Figure S2: Total amounts of polyphenols, reducing sugars, proteins and antioxidant activity of methanol extracts after spectrophotometric analysis. Figure S3: PCA analysis of methanol extracts spectrophotometric results after normalization showing the grouping of organs according to the concentrations of reducing sugars, polyphenols, proteins and antioxidant activity. Table S1: Complete dataset. Original and normalized spectrophotometric data on water and methanol extracts.

## Abbreviations

AA: ascorbic acid; ABTS: 2,2′-azino-di-(3-ethylbenzthiazoline sulfonic acid); Antiox: antioxidant activity; Antiox_Norm: normalized antioxidant activity; ba: bark; BSA: bovine serum albumin; DNS: 3,5-dinitrosalicylic acid; DW: dry weight; eq: equivalent; fl: flowers; GA: gallic acid; GLU: D-glucose; L: Liguria; le: leaves; P: Apulia; PCA: Principal Component Analysis; Poly: polyphenols; Poly_Norm: normalized polyphenols; Prot: proteins; Prot_Norm: normalized proteins; r: region; RedSug: reducing sugars; RedSug_Norm: normalized reducing sugars; ro: roots; sp: species; st: stems; T: Tuscany.
