# Supplementary material for: New Insights on Primary and Secondary Metabolite Contents of Seven Italian Wild Food Plants with Medicinal Applications: A Comparative Study
Source: Plants (Basel). 2023 Sep 5;12(18):3180. doi: 10.3390/plants12183180 (PMC10537336; doi:10.3390/plants12183180)
Supplement: Supplementary file 1 [file plants-12-03180-s001.zip › Supplementary Figures and Table Monari et al rev 1.pdf]

Article

New Insights on Primary and Secondary Metabolite Contents of Seven Italian Wild Food Plants with Medicinal Applications: A Comparative Study

Stefania Monari, Maura Ferri, Mirko Salinitro and Annalisa Tassoni *

Department of Biological, Geological and Environmental Sciences, University of Bologna, Via Irnerio n. 42, 40126 Bologna, Italy
* Correspondence: annalisa.tassoni2@unibo.it; Tel.: +39-051-2091280

Abstract: Wild food plants are widely consumed all over the world and many have both nutritional and therapeutic value due to the presence of biologically active compounds. The present research, for the first time, aims to compare primary and secondary metabolite levels among different plant organs (flower, leaf, stem, root, bark) of seven species (*Borago officinalis* L., *Cynodon dactylon* (L.) Pers., *Foeniculum vulgare* Mill., *Hypericum perforatum* L., *Malva sylvestris* L., *Sambucus nigra* L., *Urtica dioica* L.) collected in three different Italian regions (Liguria, Tuscany, Apulia). Plant organ samples were extracted with water or 95% (v/v) methanol and liquid fractions were analyzed using spectrophotometric assays. The best results were obtained for *Hypericum perforatum* L. samples, followed by *Sambucus nigra* L. and *Borago officinalis* L. As also confirmed via PCA analysis on normalized data, flower and leaf extracts of all species exhibited higher levels of polyphenols (up to 105.7 mg GA eq/gDW), reducing sugars (up to 389.2 mg GLUC eq/gDW), proteins (up to 675.7 mg BSA eq/gDW) and of antioxidant capacity (up to 263.5 mg AA eq/gDW). No differences among the regions of gathering were detected after spectrophotometric assays, which was confirmed via PCA analysis. These data contribute to further validate the traditionally reported healing effects of these species on human health.

Keywords: antioxidant activity; medicinal plants; polyphenols; wild food plants

Supplementary Materials list of contents:

Figure S1: PCA analysis of spectrophotometric results on methanol extracts, showing the grouping of species according to total polyphenols, reducing sugars, proteins and antioxidant activity data.

Figure S2: Total amounts of polyphenols, reducing sugars, proteins and antioxidant activity of methanol extracts after spectrophotometric analysis.

Figure S3: PCA analysis of methanol extracts spectrophotometric results after normalization showing the grouping of organs according to the concentrations of reducing sugars, polyphenols, proteins and antioxidant activity.

Table S1: Complete dataset. Original and normalized spectrophotometric data on water and methanol extracts.

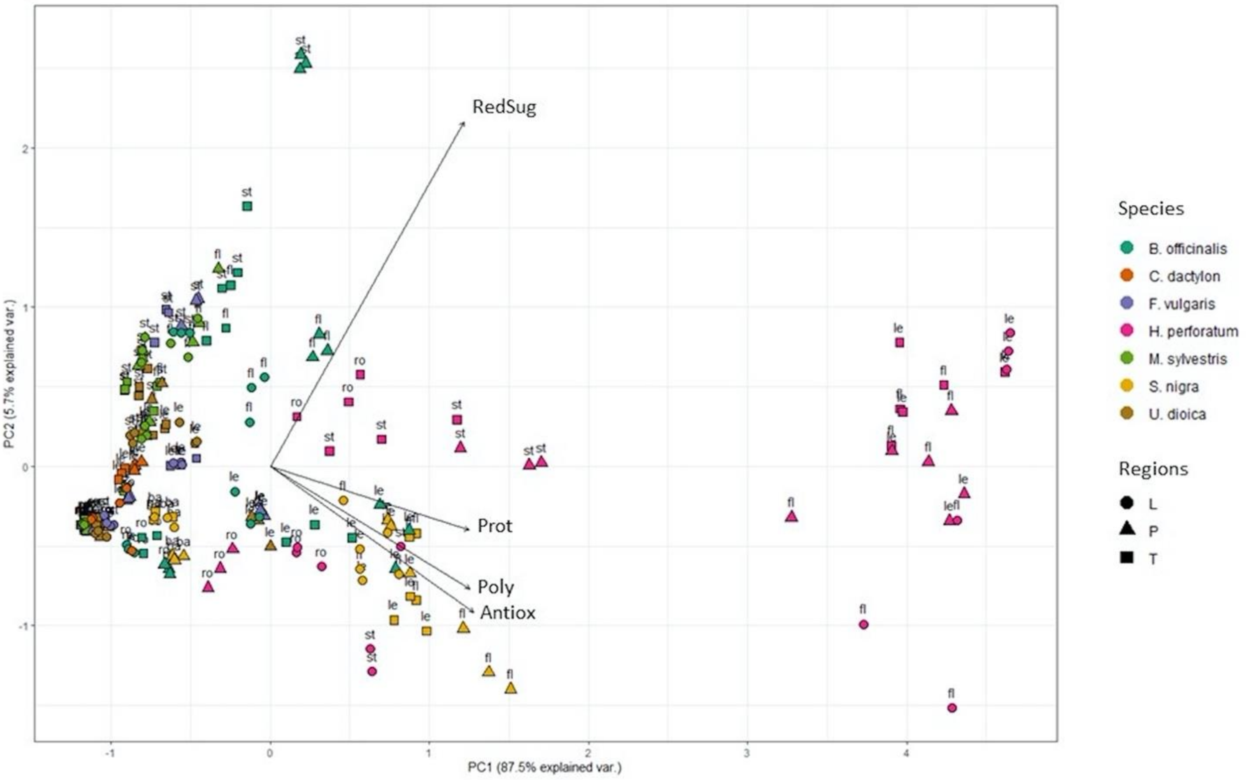

Supplementary Figure S1. PCA analysis of spectrophotometrical results on methanol extracts, showing the grouping of species according to total polyphenols, reducing sugars, proteins and antioxidant activity data (Fig. S2). L: Liguria; P: Apulia; T: Tuscany; fl: flowers; le: leaves; st: stems; ro: roots; ba: bark; RedSug: reducing sugars; Poly: polyphenols; Antiox: antioxidant activity; Prot: proteins.

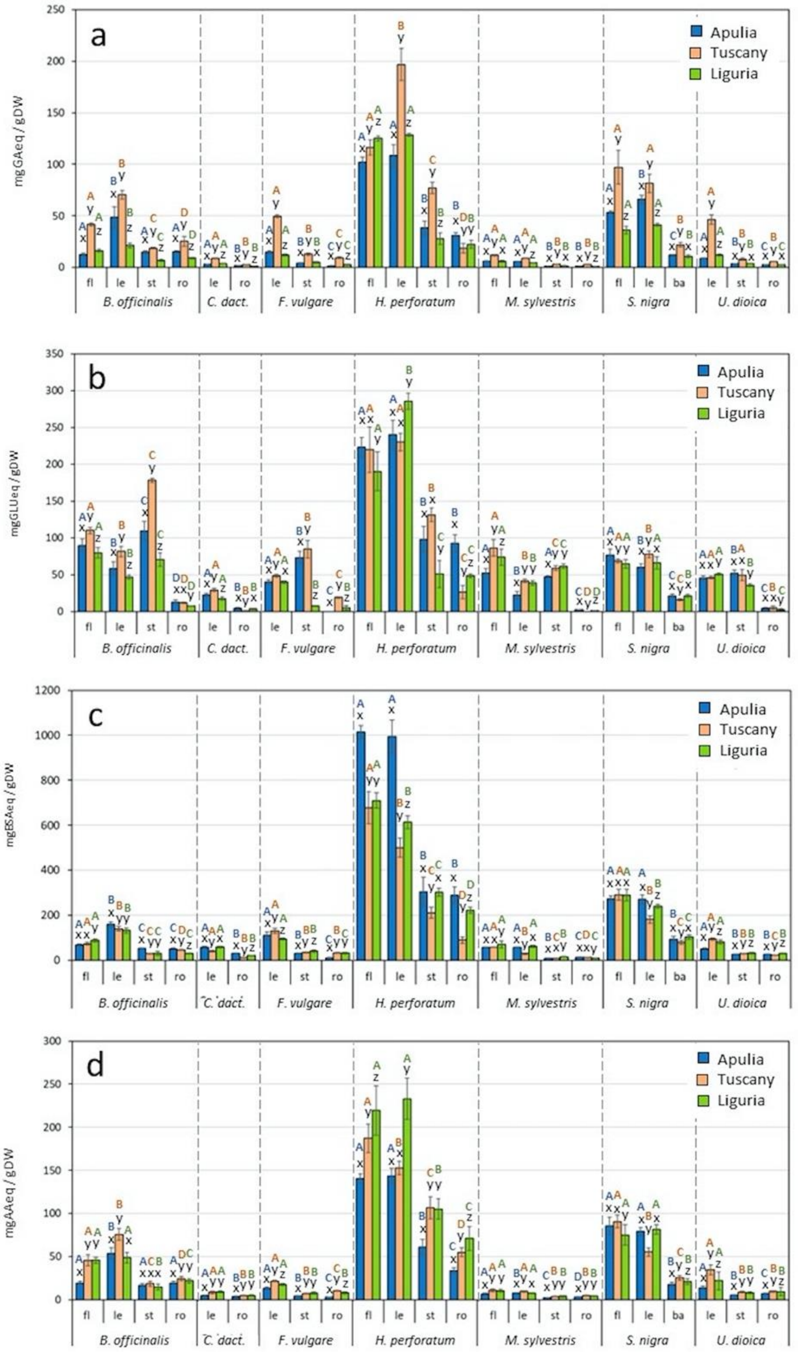

Supplementary Figure S2. Total amounts of polyphenols (a), reducing sugars (b), proteins (c) and antioxidant activity (d) of methanol extracts after spectrophotometrical analysis. fl: flowers; le: leaves; st: stems; ro: roots; ba: bark; GA: gallic acid, GLU: D-glucose, BSA: bovine serum albumin, AA: ascorbic acid, eq: equivalent, DW: dry weight. Different letters indicate a statistically significant difference (one-way ANOVA followed by post hoc Tukey HSD test or Kruskal-Wallis followed by Dunn test, $p < 0.05$) among the regions (x, y, z) and among the organs of each species (A, B, C, D). Data are the mean ± SD (n = 3).

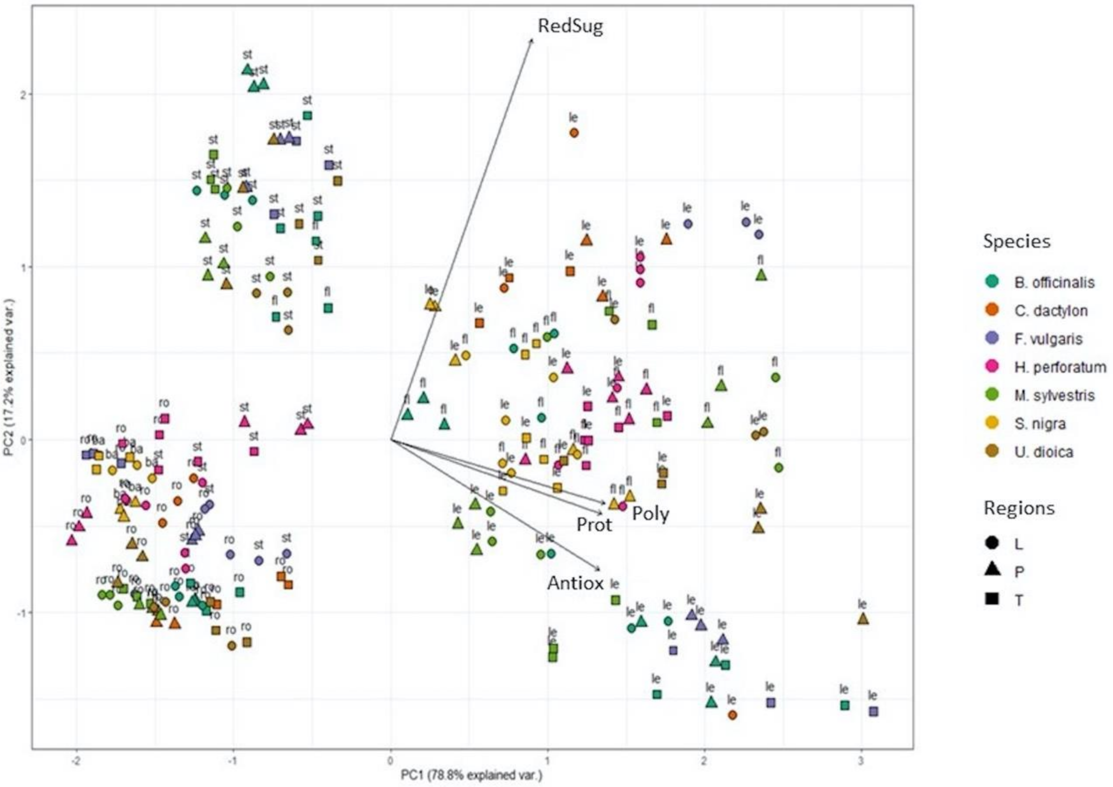

Supplementary Figure S3. PCA analysis of methanol extracts spectrophotometrical results (Table S1) after normalization (see Methods section) showing the grouping of organs according to the concentrations of reducing sugars, polyphenols, proteins and antioxidant activity. L: Liguria; P: Apulia; T: Tuscany; fl: flowers; le: leaves; st: stems; ro: roots; ba: bark; RedSug: reducing sugars; Poly: polyphenols; Antiox: antioxidant activity; Prot: proteins.

Supplementary Table S1: Complete dataset. Original and normalized (see Methods section) spectrophotometrical data on water and methanol extracts. Polyphenols (Poly), reducing sugars (RedSug), proteins (Prot) and antioxidant activity (Antiox); Poly_norm: normalized polyphenols values; RedSug_norm: normalized reducing sugars values; Prot_norm: normalized proteins values; fl: flowers; le: leaves; st: stems; ro: roots; ba: bark; L: Liguria; P: Apulia; T: Tuscany. Antiox_norm: normalized antioxidant activity values; AA: ascorbic acid; GA: gallic acid; GLU: D-glucose; BSA: bovine serum albumin; eq: equivalent, DW: dry weight.

**Species**	**Organ**	**Region**	**Solvent extraction**	**Antiox (mgAAeq/gDW)**	**Poly (mgGAeq/gDW)**	**RedSug (mgGLUeq/gDW)**	**Prot (mgBSAeq/gDW)**	**Antiox_norm**	**Poly_norm**	**RedSug_norm**	**Prot_norm**
B. officinalis	fl	P	water	74.05	15.10	137.55	163.47	0.84	0.45	0.57	0.62
B. officinalis	fl	P	water	75.49	16.34	158.57	180.19	0.85	0.49	0.66	0.68
B. officinalis	fl	P	water	62.81	17.19	149.91	196.67	0.71	0.51	0.62	0.74
B. officinalis	le	P	water	97.62	33.65	121.26	199.04	1.10	1.00	0.50	0.75
B. officinalis	le	P	water	64.53	33.97	107.60	188.83	0.73	1.01	0.45	0.71
B. officinalis	le	P	water	58.61	36.72	116.05	187.16	0.66	1.09	0.48	0.71
B. officinalis	st	P	water	19.35	7.27	174.39	62.68	0.22	0.22	0.72	0.24
B. officinalis	st	P	water	19.55	7.90	161.68	81.89	0.22	0.24	0.67	0.31
B. officinalis	st	P	water	14.07	9.00	168.01	88.45	0.16	0.27	0.70	0.33
B. officinalis	ro	P	water	17.56	10.09	66.97	105.03	0.20	0.30	0.28	0.40
B. officinalis	ro	P	water	14.35	7.09	37.97	66.22	0.16	0.21	0.16	0.25
B. officinalis	ro	P	water	13.82	7.29	47.71	69.46	0.16	0.22	0.20	0.26
B. officinalis	fl	T	water	67.76	21.58	144.78	228.30	0.78	0.59	0.58	0.71
B. officinalis	fl	T	water	50.15	22.21	148.27	183.09	0.58	0.60	0.59	0.57
B. officinalis	fl	T	water	63.18	20.96	134.09	214.25	0.73	0.57	0.53	0.66
B. officinalis	le	T	water	99.46	35.72	112.95	297.29	1.15	0.97	0.45	0.92
B. officinalis	le	T	water	80.20	34.40	118.24	278.77	0.92	0.93	0.47	0.86
B. officinalis	le	T	water	79.70	38.05	108.59	299.99	0.92	1.03	0.43	0.93
B. officinalis	st	T	water	13.73	8.84	198.07	71.11	0.16	0.24	0.79	0.22
B. officinalis	st	T	water	12.46	7.59	214.52	73.68	0.14	0.21	0.85	0.23
B. officinalis	st	T	water	13.29	7.90	234.72	63.01	0.15	0.21	0.93	0.20
B. officinalis	ro	T	water	12.79	7.68	31.79	69.59	0.15	0.21	0.13	0.22
B. officinalis	ro	T	water	13.82	7.17	29.47	73.73	0.16	0.19	0.12	0.23
B. officinalis	ro	T	water	14.46	9.07	33.02	84.99	0.17	0.25	0.13	0.26
B. officinalis	fl	L	water	49.31	17.50	131.77	163.97	0.83	0.62	0.70	0.68
B. officinalis	fl	L	water	50.00	17.70	154.86	149.68	0.84	0.63	0.83	0.62
B. officinalis	fl	L	water	51.50	19.49	143.38	183.43	0.87	0.70	0.77	0.76
B. officinalis	le	L	water	38.51	20.18	54.62	213.58	0.65	0.72	0.29	0.88
B. officinalis	le	L	water	41.75	21.81	98.36	241.46	0.70	0.78	0.53	1.00

B. officinalis	le	L	water	45.27	16.80	68.76	185.31	0.76	0.60	0.37	0.76
B. officinalis	st	L	water	16.15	13.07	155.15	59.27	0.27	0.47	0.83	0.24
B. officinalis	st	L	water	13.56	9.47	135.51	39.33	0.23	0.34	0.72	0.16
B. officinalis	st	L	water	18.14	12.40	113.35	78.73	0.31	0.44	0.61	0.32
B. officinalis	ro	L	water	12.74	6.97	22.12	44.59	0.21	0.25	0.12	0.18
B. officinalis	ro	L	water	10.72	6.28	21.71	43.70	0.18	0.22	0.12	0.18
B. officinalis	ro	L	water	8.46	6.34	22.65	52.63	0.14	0.23	0.12	0.22
C. dactylon	le	P	water	12.50	8.20	80.27	108.89	0.60	0.79	0.67	0.73
C. dactylon	le	P	water	17.04	8.01	87.91	115.60	0.82	0.77	0.73	0.78
C. dactylon	le	P	water	13.13	8.49	80.96	117.69	0.63	0.81	0.67	0.79
C. dactylon	ro	P	water	6.74	2.13	43.85	39.68	0.32	0.20	0.37	0.27
C. dactylon	ro	P	water	6.92	2.47	37.28	31.13	0.33	0.24	0.31	0.21
C. dactylon	ro	P	water	6.28	2.02	29.98	32.11	0.30	0.19	0.25	0.22
C. dactylon	le	T	water	14.70	10.71	53.68	152.11	0.70	0.80	0.63	0.82
C. dactylon	le	T	water	17.05	12.61	62.93	180.21	0.81	0.95	0.74	0.97
C. dactylon	le	T	water	14.05	10.26	46.88	144.46	0.67	0.77	0.55	0.78
C. dactylon	ro	T	water	6.83	2.15	36.96	33.22	0.32	0.16	0.43	0.18
C. dactylon	ro	T	water	5.35	2.07	28.35	15.03	0.25	0.16	0.33	0.08
C. dactylon	ro	T	water	5.13	2.19	26.17	32.72	0.24	0.16	0.31	0.18
C. dactylon	le	L	water	10.49	9.69	30.37	109.39	0.55	0.82	0.54	0.78
C. dactylon	le	L	water	11.33	8.90	27.04	107.52	0.59	0.75	0.48	0.76
C. dactylon	le	L	water	11.60	10.13	27.29	113.83	0.60	0.86	0.48	0.81
C. dactylon	ro	L	water	7.80	2.36	29.84	31.74	0.41	0.20	0.53	0.23
C. dactylon	ro	L	water	8.31	2.19	26.87	29.43	0.43	0.18	0.47	0.21
C. dactylon	ro	L	water	8.06	2.28	28.35	30.58	0.42	0.19	0.50	0.22
F. vulgaris	le	P	water	27.50	18.37	71.93	215.20	1.06	1.04	0.41	1.08
F. vulgaris	le	P	water	24.95	20.46	61.61	180.24	0.96	1.16	0.35	0.90
F. vulgaris	le	P	water	23.98	20.73	68.93	222.14	0.92	1.18	0.39	1.11
F. vulgaris	st	P	water	11.29	5.17	171.57	71.06	0.43	0.29	0.97	0.36
F. vulgaris	st	P	water	7.17	4.88	189.23	74.44	0.28	0.28	1.07	0.37
F. vulgaris	st	P	water	6.56	5.07	181.02	66.89	0.25	0.29	1.03	0.34
F. vulgaris	ro	P	water	5.60	1.56	12.88	23.51	0.22	0.09	0.07	0.12
F. vulgaris	ro	P	water	5.02	1.42	22.87	22.00	0.19	0.08	0.13	0.11
F. vulgaris	ro	P	water	4.75	1.51	13.45	22.23	0.18	0.09	0.08	0.11
F. vulgaris	le	T	water	19.76	16.45	114.74	193.80	0.83	0.94	0.45	0.94
F. vulgaris	le	T	water	14.53	15.42	126.88	170.74	0.61	0.89	0.50	0.83

F. vulgaris	le	T	water	17.55	18.93	126.47	195.49	0.74	1.09	0.50	0.95
F. vulgaris	st	T	water	8.83	6.31	215.35	82.62	0.37	0.36	0.84	0.40
F. vulgaris	st	T	water	6.36	5.80	242.82	79.93	0.27	0.33	0.95	0.39
F. vulgaris	st	T	water	5.34	5.30	192.76	82.55	0.23	0.30	0.76	0.40
F. vulgaris	ro	T	water	7.33	3.43	42.98	31.78	0.31	0.20	0.17	0.15
F. vulgaris	ro	T	water	15.48	3.36	43.28	51.01	0.65	0.19	0.17	0.25
F. vulgaris	ro	T	water	11.41	3.39	43.13	41.40	0.48	0.19	0.17	0.20
F. vulgaris	le	L	water	27.00	25.97	91.36	231.16	0.91	1.41	0.72	1.40
F. vulgaris	le	L	water	31.19	23.10	91.19	156.67	1.05	1.26	0.72	0.95
F. vulgaris	le	L	water	27.40	16.02	74.87	164.91	0.92	0.87	0.59	1.00
F. vulgaris	st	L	water	10.96	3.00	90.65	39.40	0.37	0.16	0.71	0.24
F. vulgaris	st	L	water	8.72	4.16	86.62	42.72	0.29	0.23	0.68	0.26
F. vulgaris	st	L	water	8.37	4.29	92.59	37.71	0.28	0.23	0.73	0.23
F. vulgaris	ro	L	water	7.51	2.25	19.55	23.56	0.25	0.12	0.15	0.14
F. vulgaris	ro	L	water	7.73	2.23	16.93	24.83	0.26	0.12	0.13	0.15
F. vulgaris	ro	L	water	4.47	1.70	9.76	20.22	0.15	0.09	0.08	0.12
H. perforatum	fl	P	water	230.49	72.49	309.45	503.54	0.77	0.66	0.69	0.70
H. perforatum	fl	P	water	322.08	66.53	322.63	523.78	1.07	0.61	0.72	0.73
H. perforatum	fl	P	water	208.36	79.78	295.44	466.07	0.69	0.73	0.66	0.65
H. perforatum	le	P	water	204.04	73.19	367.14	678.46	0.68	0.67	0.82	0.94
H. perforatum	le	P	water	211.67	109.82	412.32	681.57	0.70	1.00	0.93	0.94
H. perforatum	le	P	water	232.49	107.92	388.10	667.15	0.77	0.99	0.87	0.92
H. perforatum	st	P	water	75.40	26.55	113.15	140.01	0.25	0.24	0.25	0.19
H. perforatum	st	P	water	78.68	33.97	149.49	179.72	0.26	0.31	0.34	0.25
H. perforatum	st	P	water	56.20	28.78	125.85	153.97	0.19	0.26	0.28	0.21
H. perforatum	ro	P	water	64.09	18.91	62.23	118.89	0.21	0.17	0.14	0.16
H. perforatum	ro	P	water	62.66	19.72	62.96	105.98	0.21	0.18	0.14	0.15
H. perforatum	ro	P	water	56.29	19.45	63.62	109.38	0.19	0.18	0.14	0.15
H. perforatum	fl	T	water	175.02	105.47	355.13	429.29	0.79	0.91	0.97	0.88
H. perforatum	fl	T	water	180.39	104.11	337.30	331.22	0.82	0.90	0.93	0.68
H. perforatum	fl	T	water	190.45	107.39	328.52	426.09	0.86	0.93	0.90	0.87
H. perforatum	le	T	water	164.88	81.15	217.51	363.07	0.75	0.70	0.60	0.74
H. perforatum	le	T	water	141.79	91.08	235.02	398.03	0.64	0.79	0.64	0.82
H. perforatum	le	T	water	92.70	95.44	228.06	409.53	0.42	0.82	0.63	0.84
H. perforatum	st	T	water	71.80	29.48	129.64	147.31	0.33	0.25	0.36	0.30
H. perforatum	st	T	water	90.78	28.30	136.94	166.05	0.41	0.24	0.38	0.34

H. perforatum	st	T	water	86.37	29.56	134.86	139.51	0.39	0.26	0.37	0.29
H. perforatum	ro	T	water	45.79	7.77	27.26	38.05	0.21	0.07	0.07	0.08
H. perforatum	ro	T	water	39.26	7.79	28.82	41.07	0.18	0.07	0.08	0.08
H. perforatum	ro	T	water	42.52	7.78	28.04	39.56	0.19	0.07	0.08	0.08
H. perforatum	fl	L	water	178.92	85.31	276.28	526.01	0.58	0.84	0.82	0.92
H. perforatum	fl	L	water	258.92	71.31	308.18	462.28	0.84	0.70	0.91	0.81
H. perforatum	fl	L	water	217.21	80.72	300.63	402.78	0.71	0.80	0.89	0.70
H. perforatum	le	L	water	318.03	95.65	254.76	524.74	1.04	0.94	0.75	0.92
H. perforatum	le	L	water	200.30	107.10	260.85	569.02	0.65	1.06	0.77	1.00
H. perforatum	le	L	water	272.08	85.99	275.49	476.65	0.89	0.85	0.81	0.83
H. perforatum	st	L	water	106.93	21.18	93.72	119.82	0.35	0.21	0.28	0.21
H. perforatum	st	L	water	72.72	15.00	69.40	85.66	0.24	0.15	0.20	0.15
H. perforatum	st	L	water	95.81	17.47	84.83	103.84	0.31	0.17	0.25	0.18
H. perforatum	ro	L	water	31.49	10.61	37.86	77.60	0.10	0.10	0.11	0.14
H. perforatum	ro	L	water	45.99	11.81	34.98	46.59	0.15	0.12	0.10	0.08
H. perforatum	ro	L	water	43.90	6.15	35.22	35.13	0.14	0.06	0.10	0.06
M. sylvestris	fl	P	water	29.52	16.24	107.65	156.28	0.60	0.75	0.73	0.73
M. sylvestris	fl	P	water	45.49	17.18	125.16	195.35	0.93	0.79	0.85	0.91
M. sylvestris	fl	P	water	37.52	18.10	106.58	177.44	0.77	0.84	0.72	0.83
M. sylvestris	le	P	water	49.28	22.63	53.44	202.79	1.01	1.05	0.36	0.95
M. sylvestris	le	P	water	39.65	16.51	29.93	184.88	0.81	0.76	0.20	0.87
M. sylvestris	le	P	water	35.24	14.14	18.16	157.07	0.72	0.65	0.12	0.73
M. sylvestris	st	P	water	14.06	5.13	123.71	29.03	0.29	0.24	0.84	0.14
M. sylvestris	st	P	water	9.81	6.09	124.65	49.28	0.20	0.28	0.84	0.23
M. sylvestris	st	P	water	11.73	5.83	116.06	45.06	0.24	0.27	0.78	0.21
M. sylvestris	ro	P	water	7.62	2.65	28.12	28.45	0.16	0.12	0.19	0.13
M. sylvestris	ro	P	water	6.78	2.56	26.59	28.29	0.14	0.12	0.18	0.13
M. sylvestris	ro	P	water	7.20	2.61	27.35	28.37	0.15	0.12	0.18	0.13
M. sylvestris	fl	T	water	31.54	16.14	189.95	158.52	0.73	0.75	1.13	0.71
M. sylvestris	fl	T	water	31.16	17.17	159.29	174.22	0.73	0.79	0.95	0.78
M. sylvestris	fl	T	water	26.93	15.24	150.59	173.73	0.63	0.70	0.90	0.78
M. sylvestris	le	T	water	29.80	21.76	42.63	237.47	0.69	1.01	0.25	1.07
M. sylvestris	le	T	water	52.96	20.43	42.38	218.50	1.23	0.95	0.25	0.98
M. sylvestris	le	T	water	44.55	18.47	37.66	131.48	1.04	0.85	0.22	0.59
M. sylvestris	st	T	water	7.62	4.26	109.20	46.25	0.18	0.20	0.65	0.21
M. sylvestris	st	T	water	9.22	4.50	112.30	53.76	0.21	0.21	0.67	0.24

M. sylvestris	st	T	water	8.45	4.56	103.38	44.98	0.20	0.21	0.61	0.20
M. sylvestris	ro	T	water	5.84	2.57	20.81	36.73	0.14	0.12	0.12	0.16
M. sylvestris	ro	T	water	4.94	2.10	21.30	35.90	0.11	0.10	0.13	0.16
M. sylvestris	ro	T	water	4.70	2.49	19.28	25.71	0.11	0.12	0.11	0.12
M. sylvestris	fl	L	water	23.02	21.74	128.63	222.72	0.58	0.76	0.88	0.79
M. sylvestris	fl	L	water	28.61	21.61	129.35	210.82	0.72	0.76	0.88	0.74
M. sylvestris	fl	L	water	37.39	23.12	116.43	209.18	0.94	0.81	0.79	0.74
M. sylvestris	le	L	water	31.37	28.79	59.67	268.88	0.79	1.01	0.41	0.95
M. sylvestris	le	L	water	42.28	29.48	62.95	329.04	1.07	1.04	0.43	1.16
M. sylvestris	le	L	water	37.18	26.30	67.40	281.34	0.94	0.92	0.46	0.99
M. sylvestris	st	L	water	6.56	4.54	99.08	46.07	0.17	0.16	0.68	0.16
M. sylvestris	st	L	water	9.21	4.19	101.37	34.07	0.23	0.15	0.69	0.12
M. sylvestris	st	L	water	7.99	4.90	72.58	38.82	0.20	0.17	0.49	0.14
M. sylvestris	ro	L	water	3.33	2.23	16.25	20.90	0.08	0.08	0.11	0.07
M. sylvestris	ro	L	water	5.10	1.72	10.80	17.38	0.13	0.06	0.07	0.06
M. sylvestris	ro	L	water	5.65	2.12	15.53	19.16	0.14	0.07	0.11	0.07
S. nigra	fl	P	water	73.53	36.78	201.87	358.22	0.52	0.42	0.71	0.61
S. nigra	fl	P	water	68.02	33.53	175.96	367.70	0.48	0.38	0.62	0.63
S. nigra	fl	P	water	77.86	40.32	167.38	376.49	0.55	0.46	0.59	0.64
S. nigra	le	P	water	112.19	72.48	153.83	367.79	0.80	0.82	0.54	0.63
S. nigra	le	P	water	103.66	81.74	173.62	365.08	0.73	0.92	0.61	0.62
S. nigra	le	P	water	117.80	86.63	175.14	416.39	0.84	0.98	0.61	0.71
S. nigra	ba	P	water	28.29	15.35	72.89	135.51	0.20	0.17	0.26	0.23
S. nigra	ba	P	water	27.30	17.19	77.79	115.89	0.19	0.19	0.27	0.20
S. nigra	ba	P	water	26.02	14.58	86.63	141.39	0.18	0.16	0.30	0.24
S. nigra	fl	T	water	71.20	31.01	188.07	156.21	0.53	0.47	0.67	0.53
S. nigra	fl	T	water	123.91	37.91	182.84	161.69	0.92	0.57	0.65	0.55
S. nigra	fl	T	water	102.75	36.20	162.35	166.74	0.77	0.55	0.57	0.57
S. nigra	le	T	water	81.43	50.08	186.73	119.57	0.61	0.76	0.66	0.41
S. nigra	le	T	water	61.34	51.39	186.93	135.89	0.46	0.78	0.66	0.46
S. nigra	le	T	water	83.42	45.97	184.66	114.66	0.62	0.70	0.65	0.39
S. nigra	ba	T	water	28.20	14.84	60.46	166.38	0.21	0.22	0.21	0.57
S. nigra	ba	T	water	25.77	15.72	59.92	134.78	0.19	0.24	0.21	0.46
S. nigra	ba	T	water	26.23	14.04	60.47	167.88	0.20	0.21	0.21	0.57
S. nigra	fl	L	water	79.90	28.78	100.86	249.13	0.79	0.53	0.45	0.60
S. nigra	fl	L	water	63.65	27.19	120.62	182.63	0.63	0.50	0.53	0.44

S. nigra	fl	L	water	64.14	26.28	102.97	242.80	0.64	0.49	0.45	0.59
S. nigra	le	L	water	52.54	47.99	197.27	284.18	0.52	0.89	0.87	0.69
S. nigra	le	L	water	65.77	44.08	172.96	277.65	0.65	0.82	0.76	0.67
S. nigra	le	L	water	67.46	41.02	193.59	275.82	0.67	0.76	0.86	0.67
S. nigra	ba	L	water	19.91	10.14	46.68	125.71	0.20	0.19	0.21	0.30
S. nigra	ba	L	water	18.69	8.72	42.81	127.97	0.19	0.16	0.19	0.31
S. nigra	ba	L	water	21.73	8.52	41.09	100.20	0.22	0.16	0.18	0.24
U. dioica	le	P	water	55.12	12.85	49.77	178.77	1.19	0.89	0.42	0.93
U. dioica	le	P	water	56.87	14.40	60.51	148.38	1.23	1.00	0.52	0.77
U. dioica	le	P	water	42.71	13.26	50.83	197.43	0.92	0.92	0.43	1.02
U. dioica	st	P	water	10.42	5.05	71.07	67.56	0.22	0.35	0.61	0.35
U. dioica	st	P	water	9.01	4.88	78.94	64.17	0.19	0.34	0.67	0.33
U. dioica	st	P	water	7.29	4.73	72.89	63.35	0.16	0.33	0.62	0.33
U. dioica	ro	P	water	7.85	3.11	42.55	51.21	0.17	0.22	0.36	0.27
U. dioica	ro	P	water	10.03	3.20	52.99	62.55	0.22	0.22	0.45	0.32
U. dioica	ro	P	water	9.27	3.64	48.99	36.05	0.20	0.25	0.42	0.19
U. dioica	le	T	water	64.49	16.92	116.29	269.13	1.16	0.94	0.64	1.01
U. dioica	le	T	water	65.45	18.97	135.51	232.14	1.18	1.05	0.74	0.87
U. dioica	le	T	water	66.27	19.62	129.77	275.73	1.20	1.09	0.71	1.04
U. dioica	st	T	water	9.62	5.38	112.67	75.41	0.17	0.30	0.62	0.28
U. dioica	st	T	water	7.03	4.46	101.98	65.07	0.13	0.25	0.56	0.24
U. dioica	st	T	water	9.31	4.68	94.77	65.71	0.17	0.26	0.52	0.25
U. dioica	ro	T	water	9.28	4.09	45.56	74.74	0.17	0.23	0.25	0.28
U. dioica	ro	T	water	7.40	3.30	45.04	72.17	0.13	0.18	0.25	0.27
U. dioica	ro	T	water	10.33	3.53	38.71	66.64	0.19	0.20	0.21	0.25
U. dioica	le	L	water	59.01	16.42	61.48	195.62	1.26	1.01	0.72	0.93
U. dioica	le	L	water	38.77	17.86	64.33	173.65	0.83	1.10	0.76	0.83
U. dioica	le	L	water	57.56	15.86	57.27	206.94	1.23	0.97	0.67	0.99
U. dioica	st	L	water	9.90	4.71	49.97	51.52	0.21	0.29	0.59	0.25
U. dioica	st	L	water	6.50	4.43	41.53	56.98	0.14	0.27	0.49	0.27
U. dioica	st	L	water	8.73	4.67	43.80	64.07	0.19	0.29	0.51	0.31
U. dioica	ro	L	water	12.71	3.21	18.57	62.11	0.27	0.20	0.22	0.30
U. dioica	ro	L	water	7.82	2.81	23.37	63.71	0.17	0.17	0.27	0.30
U. dioica	ro	L	water	9.70	3.29	22.59	70.20	0.21	0.20	0.27	0.33
B. officinalis	fl	P	methanol	18.29	10.91	79.77	64.00	0.33	0.24	0.59	0.39
B. officinalis	fl	P	methanol	19.01	12.55	100.50	68.63	0.35	0.27	0.74	0.41

B. officinalis	fl	P	methanol	21.36	14.32	88.08	72.67	0.39	0.31	0.65	0.44
B. officinalis	le	P	methanol	59.65	58.49	67.01	172.94	1.09	1.28	0.50	1.04
B. officinalis	le	P	methanol	52.29	38.41	48.57	147.23	0.96	0.84	0.36	0.89
B. officinalis	le	P	methanol	49.58	49.06	60.14	160.42	0.91	1.07	0.44	0.97
B. officinalis	st	P	methanol	16.01	13.43	125.62	50.91	0.29	0.29	0.93	0.31
B. officinalis	st	P	methanol	14.18	15.02	97.54	53.93	0.26	0.33	0.72	0.33
B. officinalis	st	P	methanol	19.21	16.03	106.03	53.15	0.35	0.35	0.78	0.32
B. officinalis	ro	P	methanol	21.33	16.16	16.99	57.13	0.39	0.35	0.13	0.34
B. officinalis	ro	P	methanol	19.29	15.39	8.91	49.42	0.35	0.34	0.07	0.30
B. officinalis	ro	P	methanol	17.75	14.11	12.67	44.79	0.32	0.31	0.09	0.27
B. officinalis	fl	T	methanol	42.60	43.13	105.48	67.84	0.52	0.55	0.55	0.47
B. officinalis	fl	T	methanol	42.24	41.39	112.81	78.10	0.51	0.53	0.59	0.54
B. officinalis	fl	T	methanol	52.14	40.47	111.47	73.23	0.63	0.52	0.58	0.51
B. officinalis	le	T	methanol	70.74	64.80	85.18	128.66	0.86	0.83	0.45	0.89
B. officinalis	le	T	methanol	78.90	73.44	86.32	142.87	0.96	0.94	0.45	0.99
B. officinalis	le	T	methanol	77.64	72.90	72.71	150.17	0.94	0.93	0.38	1.04
B. officinalis	st	T	methanol	16.81	18.45	179.67	28.23	0.20	0.24	0.94	0.19
B. officinalis	st	T	methanol	20.85	18.59	179.07	29.95	0.25	0.24	0.94	0.21
B. officinalis	st	T	methanol	18.01	18.82	175.74	31.02	0.22	0.24	0.92	0.21
B. officinalis	ro	T	methanol	24.05	26.35	12.30	46.49	0.29	0.34	0.06	0.32
B. officinalis	ro	T	methanol	22.93	28.73	11.17	44.48	0.28	0.37	0.06	0.31
B. officinalis	ro	T	methanol	26.70	21.08	12.25	48.21	0.32	0.27	0.06	0.33
B. officinalis	fl	L	methanol	47.45	16.94	86.91	85.66	0.72	0.63	0.83	0.62
B. officinalis	fl	L	methanol	45.15	14.56	80.68	83.52	0.69	0.54	0.77	0.61
B. officinalis	fl	L	methanol	44.83	17.12	70.92	96.67	0.68	0.64	0.68	0.70
B. officinalis	le	L	methanol	51.35	21.78	43.98	134.26	0.78	0.81	0.42	0.97
B. officinalis	le	L	methanol	50.67	23.76	47.71	144.73	0.77	0.89	0.45	1.05
B. officinalis	le	L	methanol	44.00	18.34	48.48	116.97	0.67	0.68	0.46	0.85
B. officinalis	st	L	methanol	12.01	6.18	74.33	21.09	0.18	0.23	0.71	0.15
B. officinalis	st	L	methanol	17.88	8.00	78.45	27.56	0.27	0.30	0.75	0.20
B. officinalis	st	L	methanol	14.94	7.09	76.39	24.32	0.23	0.26	0.73	0.18
B. officinalis	ro	L	methanol	19.07	9.18	7.37	31.22	0.29	0.34	0.07	0.23
B. officinalis	ro	L	methanol	22.35	8.48	6.92	29.72	0.34	0.32	0.07	0.22
B. officinalis	ro	L	methanol	24.36	9.64	7.72	32.05	0.37	0.36	0.07	0.23
C. dactylon	le	P	methanol	5.80	3.36	24.68	55.45	0.70	0.66	0.94	0.62
C. dactylon	le	P	methanol	4.08	3.26	22.31	60.46	0.49	0.64	0.85	0.67

C. dactylon	le	P	methanol	4.09	2.97	19.82	58.67	0.50	0.58	0.75	0.65
C. dactylon	ro	P	methanol	3.96	1.96	5.18	31.23	0.48	0.38	0.20	0.35
C. dactylon	ro	P	methanol	3.91	2.03	4.94	33.98	0.47	0.40	0.19	0.38
C. dactylon	ro	P	methanol	2.90	1.75	2.18	29.38	0.35	0.34	0.08	0.33
C. dactylon	le	T	methanol	6.71	9.32	28.71	40.61	0.50	0.81	0.96	0.76
C. dactylon	le	T	methanol	9.40	9.34	31.54	43.36	0.71	0.81	1.06	0.82
C. dactylon	le	T	methanol	9.70	8.11	27.52	37.06	0.73	0.70	0.92	0.70
C. dactylon	ro	T	methanol	4.42	2.68	0.78	12.51	0.33	0.23	0.03	0.24
C. dactylon	ro	T	methanol	5.13	2.52	0.82	13.80	0.39	0.22	0.03	0.26
C. dactylon	ro	T	methanol	4.60	2.63	0.28	12.14	0.35	0.23	0.01	0.23
C. dactylon	le	L	methanol	23.35	9.06	7.32	30.88	1.22	1.30	0.41	0.45
C. dactylon	le	L	methanol	9.73	3.52	14.72	53.63	0.51	0.50	0.83	0.77
C. dactylon	le	L	methanol	9.12	3.84	19.95	61.30	0.48	0.55	1.13	0.88
C. dactylon	ro	L	methanol	5.47	1.57	4.60	21.14	0.29	0.22	0.26	0.31
C. dactylon	ro	L	methanol	4.76	1.46	2.82	20.17	0.25	0.21	0.16	0.29
C. dactylon	ro	L	methanol	5.12	1.51	3.71	20.66	0.27	0.22	0.21	0.30
F. vulgaris	le	P	methanol	10.93	14.03	36.56	94.42	0.81	1.01	0.41	0.93
F. vulgaris	le	P	methanol	14.19	14.07	39.18	106.85	1.05	1.02	0.44	1.05
F. vulgaris	le	P	methanol	14.10	16.77	44.25	130.22	1.04	1.21	0.50	1.28
F. vulgaris	st	P	methanol	3.68	4.30	77.27	30.36	0.27	0.31	0.88	0.30
F. vulgaris	st	P	methanol	4.74	4.61	77.16	33.42	0.35	0.33	0.87	0.33
F. vulgaris	st	P	methanol	3.96	3.97	65.75	28.63	0.29	0.29	0.74	0.28
F. vulgaris	ro	P	methanol	3.39	1.81	19.92	14.35	0.25	0.13	0.23	0.14
F. vulgaris	ro	P	methanol	2.82	1.32	18.21	8.53	0.21	0.10	0.21	0.08
F. vulgaris	ro	P	methanol	2.95	1.40	19.06	9.47	0.22	0.10	0.22	0.09
F. vulgaris	le	T	methanol	21.73	49.10	47.84	141.55	0.84	1.02	0.47	1.07
F. vulgaris	le	T	methanol	21.48	50.80	49.18	119.69	0.83	1.06	0.48	0.91
F. vulgaris	le	T	methanol	21.64	48.70	48.51	130.22	0.83	1.02	0.48	0.99
F. vulgaris	st	T	methanol	7.41	13.98	88.89	35.87	0.28	0.29	0.88	0.27
F. vulgaris	st	T	methanol	6.99	13.33	87.70	36.02	0.27	0.28	0.86	0.27
F. vulgaris	st	T	methanol	6.71	11.39	77.31	31.73	0.26	0.24	0.76	0.24
F. vulgaris	ro	T	methanol	10.15	10.04	19.92	34.44	0.39	0.21	0.20	0.26
F. vulgaris	ro	T	methanol	10.61	8.99	18.21	31.92	0.41	0.19	0.18	0.24
F. vulgaris	ro	T	methanol	10.38	9.52	19.06	33.18	0.40	0.20	0.19	0.25
F. vulgaris	le	L	methanol	18.54	12.18	40.67	95.73	0.82	0.92	1.16	0.86
F. vulgaris	le	L	methanol	16.67	11.28	38.61	89.44	0.74	0.85	1.10	0.80

F. vulgaris	le	L	methanol	18.27	13.10	40.17	97.14	0.81	0.99	1.15	0.87
F. vulgaris	st	L	methanol	6.32	4.20	7.94	35.00	0.28	0.32	0.23	0.31
F. vulgaris	st	L	methanol	8.61	5.68	7.71	47.88	0.38	0.43	0.22	0.43
F. vulgaris	st	L	methanol	8.74	4.99	6.67	41.38	0.39	0.38	0.19	0.37
F. vulgaris	ro	L	methanol	8.60	2.72	8.24	31.01	0.38	0.21	0.24	0.28
F. vulgaris	ro	L	methanol	9.51	3.32	6.53	36.81	0.42	0.25	0.19	0.33
F. vulgaris	ro	L	methanol	6.38	2.26	1.06	27.65	0.28	0.17	0.03	0.25
H. perforatum	fl	P	methanol	143.78	99.20	210.41	987.04	0.76	0.71	0.64	0.76
H. perforatum	fl	P	methanol	133.98	100.91	220.49	1014.63	0.71	0.72	0.67	0.78
H. perforatum	fl	P	methanol	143.48	106.16	238.00	1041.13	0.76	0.76	0.73	0.80
H. perforatum	le	P	methanol	144.95	98.71	241.94	910.37	0.77	0.70	0.74	0.70
H. perforatum	le	P	methanol	133.98	105.11	220.95	999.47	0.71	0.75	0.68	0.77
H. perforatum	le	P	methanol	150.89	121.49	257.01	1073.39	0.80	0.87	0.79	0.82
H. perforatum	st	P	methanol	51.66	30.63	80.13	238.71	0.27	0.22	0.25	0.18
H. perforatum	st	P	methanol	72.28	45.73	118.29	383.71	0.38	0.33	0.36	0.29
H. perforatum	st	P	methanol	58.53	38.93	95.52	293.89	0.31	0.28	0.29	0.23
H. perforatum	ro	P	methanol	29.44	27.76	78.86	239.62	0.16	0.20	0.24	0.18
H. perforatum	ro	P	methanol	36.49	32.57	94.24	311.44	0.19	0.23	0.29	0.24
H. perforatum	ro	P	methanol	36.02	33.04	104.11	318.56	0.19	0.24	0.32	0.24
H. perforatum	fl	T	methanol	169.00	106.39	181.31	592.98	0.67	0.52	0.60	0.80
H. perforatum	fl	T	methanol	191.79	121.84	247.30	749.32	0.76	0.60	0.81	1.01
H. perforatum	fl	T	methanol	200.84	120.90	230.69	691.74	0.80	0.59	0.76	0.94
H. perforatum	le	T	methanol	153.71	209.46	236.42	506.56	0.61	1.02	0.78	0.69
H. perforatum	le	T	methanol	158.03	202.88	225.79	509.49	0.63	0.99	0.74	0.69
H. perforatum	le	T	methanol	146.96	178.19	228.30	483.77	0.59	0.87	0.75	0.65
H. perforatum	st	T	methanol	90.02	70.63	120.71	177.49	0.36	0.35	0.40	0.24
H. perforatum	st	T	methanol	116.74	76.35	134.60	225.61	0.47	0.37	0.44	0.31
H. perforatum	st	T	methanol	113.44	84.01	138.22	227.22	0.45	0.41	0.46	0.31
H. perforatum	ro	T	methanol	58.78	14.87	18.71	77.41	0.23	0.07	0.06	0.10
H. perforatum	ro	T	methanol	51.61	22.21	33.98	101.87	0.21	0.11	0.11	0.14
H. perforatum	ro	T	methanol	55.20	18.54	26.34	89.64	0.22	0.09	0.09	0.12
H. perforatum	fl	L	methanol	246.80	124.62	174.64	743.25	0.79	0.82	0.61	0.80
H. perforatum	fl	L	methanol	219.01	126.07	223.92	708.17	0.70	0.83	0.78	0.77
H. perforatum	fl	L	methanol	192.32	124.30	171.58	677.44	0.61	0.82	0.60	0.73
H. perforatum	le	L	methanol	234.15	127.28	279.77	628.08	0.74	0.84	0.97	0.68
H. perforatum	le	L	methanol	231.86	129.87	291.10	598.74	0.74	0.85	1.01	0.65

H. perforatum	le	L	methanol	233.00	128.58	285.44	613.41	0.74	0.85	0.99	0.66
H. perforatum	st	L	methanol	96.37	34.77	75.51	288.08	0.31	0.23	0.26	0.31
H. perforatum	st	L	methanol	114.19	21.90	35.77	316.72	0.36	0.14	0.12	0.34
H. perforatum	st	L	methanol	105.28	27.00	41.14	302.40	0.33	0.18	0.14	0.33
H. perforatum	ro	L	methanol	66.55	23.35	47.09	211.03	0.21	0.15	0.16	0.23
H. perforatum	ro	L	methanol	75.37	26.54	49.92	233.82	0.24	0.17	0.17	0.25
H. perforatum	ro	L	methanol	70.96	17.47	48.51	222.43	0.23	0.11	0.17	0.24
M. sylvestris	fl	P	methanol	5.50	6.01	54.27	57.16	0.56	0.82	0.87	0.85
M. sylvestris	fl	P	methanol	6.89	6.25	56.36	55.21	0.70	0.85	0.91	0.82
M. sylvestris	fl	P	methanol	7.06	6.48	46.94	59.59	0.72	0.88	0.76	0.88
M. sylvestris	le	P	methanol	6.93	5.78	18.64	55.60	0.70	0.79	0.30	0.82
M. sylvestris	le	P	methanol	7.91	5.91	28.28	58.46	0.80	0.80	0.46	0.86
M. sylvestris	le	P	methanol	7.32	5.34	20.05	55.53	0.74	0.73	0.32	0.82
M. sylvestris	st	P	methanol	2.39	1.53	48.93	8.64	0.24	0.21	0.79	0.13
M. sylvestris	st	P	methanol	2.80	1.43	46.03	8.60	0.28	0.19	0.74	0.13
M. sylvestris	st	P	methanol	2.45	1.49	46.42	9.44	0.25	0.20	0.75	0.14
M. sylvestris	ro	P	methanol	3.07	1.27	2.16	10.97	0.31	0.17	0.03	0.16
M. sylvestris	ro	P	methanol	3.57	1.34	2.43	13.99	0.36	0.18	0.04	0.21
M. sylvestris	ro	P	methanol	3.32	1.30	2.29	12.48	0.34	0.18	0.04	0.18
M. sylvestris	fl	T	methanol	10.72	12.50	101.23	58.76	0.72	0.92	1.07	1.09
M. sylvestris	fl	T	methanol	11.08	11.47	75.51	57.76	0.75	0.85	0.80	1.07
M. sylvestris	fl	T	methanol	11.31	11.65	82.33	56.86	0.76	0.86	0.87	1.05
M. sylvestris	le	T	methanol	10.77	8.70	39.76	28.16	0.73	0.64	0.42	0.52
M. sylvestris	le	T	methanol	9.57	9.01	41.16	27.20	0.65	0.67	0.44	0.50
M. sylvestris	le	T	methanol	8.50	9.30	44.12	33.66	0.57	0.69	0.47	0.62
M. sylvestris	st	T	methanol	3.69	3.16	61.21	7.90	0.25	0.23	0.65	0.15
M. sylvestris	st	T	methanol	4.13	3.10	56.26	8.71	0.28	0.23	0.59	0.16
M. sylvestris	st	T	methanol	4.29	3.30	59.30	9.85	0.29	0.24	0.63	0.18
M. sylvestris	ro	T	methanol	4.96	3.18	2.16	13.19	0.34	0.23	0.02	0.24
M. sylvestris	ro	T	methanol	5.10	2.55	2.43	9.86	0.34	0.19	0.03	0.18
M. sylvestris	ro	T	methanol	4.65	3.20	2.29	12.48	0.31	0.24	0.02	0.23
M. sylvestris	fl	L	methanol	12.00	6.85	82.41	73.95	0.90	1.02	0.94	0.94
M. sylvestris	fl	L	methanol	11.44	6.68	69.42	88.02	0.85	1.00	0.79	1.11
M. sylvestris	fl	L	methanol	8.13	4.95	69.48	50.76	0.61	0.74	0.80	0.64
M. sylvestris	le	L	methanol	8.42	4.99	38.82	63.47	0.63	0.75	0.44	0.80
M. sylvestris	le	L	methanol	7.28	4.41	36.42	62.53	0.54	0.66	0.42	0.79

M. sylvestris	le	L	methanol	7.53	4.33	40.88	58.77	0.56	0.65	0.47	0.74
M. sylvestris	st	L	methanol	3.29	1.52	65.06	15.16	0.25	0.23	0.74	0.19
M. sylvestris	st	L	methanol	3.82	1.51	60.98	17.36	0.29	0.23	0.70	0.22
M. sylvestris	st	L	methanol	5.44	1.87	57.71	15.35	0.41	0.28	0.66	0.19
M. sylvestris	ro	L	methanol	4.21	1.06	1.39	9.82	0.31	0.16	0.02	0.12
M. sylvestris	ro	L	methanol	4.20	0.96	0.80	8.56	0.31	0.14	0.01	0.11
M. sylvestris	ro	L	methanol	4.66	1.02	0.92	10.30	0.35	0.15	0.01	0.13
S. nigra	fl	P	methanol	85.38	53.53	83.65	258.93	0.70	0.61	0.79	0.61
S. nigra	fl	P	methanol	77.38	54.40	79.74	276.77	0.63	0.62	0.76	0.65
S. nigra	fl	P	methanol	94.63	52.22	66.02	285.90	0.78	0.60	0.63	0.67
S. nigra	le	P	methanol	78.69	66.26	65.66	254.52	0.65	0.76	0.62	0.60
S. nigra	le	P	methanol	82.69	70.15	60.08	291.58	0.68	0.80	0.57	0.69
S. nigra	le	P	methanol	76.54	61.96	54.61	267.80	0.63	0.71	0.52	0.63
S. nigra	ba	P	methanol	15.22	12.06	18.59	87.98	0.12	0.14	0.18	0.21
S. nigra	ba	P	methanol	20.94	12.56	24.08	109.91	0.17	0.14	0.23	0.26
S. nigra	ba	P	methanol	16.96	11.10	21.33	81.94	0.14	0.13	0.20	0.19
S. nigra	fl	T	methanol	89.53	85.14	71.95	268.59	0.78	0.64	0.66	0.73
S. nigra	fl	T	methanol	89.50	111.90	68.07	297.79	0.78	0.84	0.63	0.81
S. nigra	fl	T	methanol	93.22	94.47	66.29	307.95	0.82	0.71	0.61	0.84
S. nigra	le	T	methanol	60.96	72.22	81.41	168.58	0.53	0.54	0.75	0.46
S. nigra	le	T	methanol	52.55	92.17	72.01	200.40	0.46	0.69	0.66	0.54
S. nigra	le	T	methanol	52.65	79.94	79.92	174.97	0.46	0.60	0.74	0.48
S. nigra	ba	T	methanol	23.57	24.73	17.87	89.44	0.21	0.18	0.16	0.24
S. nigra	ba	T	methanol	25.69	20.45	15.79	72.95	0.22	0.15	0.15	0.20
S. nigra	ba	T	methanol	26.59	20.77	14.97	76.16	0.23	0.16	0.14	0.21
S. nigra	fl	L	methanol	61.31	32.51	70.57	266.20	0.52	0.55	0.69	0.63
S. nigra	fl	L	methanol	86.08	40.82	66.06	323.30	0.73	0.70	0.65	0.77
S. nigra	fl	L	methanol	77.80	35.26	57.97	279.84	0.66	0.60	0.57	0.66
S. nigra	le	L	methanol	85.52	42.66	76.63	241.73	0.72	0.73	0.75	0.57
S. nigra	le	L	methanol	75.10	40.92	64.59	239.82	0.63	0.70	0.64	0.57
S. nigra	le	L	methanol	84.18	39.91	57.70	238.36	0.71	0.68	0.57	0.56
S. nigra	ba	L	methanol	22.88	12.44	21.82	115.82	0.19	0.21	0.21	0.27
S. nigra	ba	L	methanol	17.55	9.27	19.44	92.43	0.15	0.16	0.19	0.22
S. nigra	ba	L	methanol	22.83	9.94	22.80	102.04	0.19	0.17	0.22	0.24
U. dioica	le	P	methanol	11.33	8.87	41.54	45.22	0.65	0.87	0.61	0.65
U. dioica	le	P	methanol	14.69	9.05	46.13	55.02	0.84	0.89	0.68	0.80

U. dioica	le	P	methanol	15.49	9.21	47.85	49.70	0.89	0.90	0.71	0.72
U. dioica	st	P	methanol	5.95	3.60	48.13	26.11	0.34	0.35	0.71	0.38
U. dioica	st	P	methanol	5.34	3.67	57.36	28.36	0.31	0.36	0.85	0.41
U. dioica	st	P	methanol	4.89	3.29	50.50	25.74	0.28	0.32	0.75	0.37
U. dioica	ro	P	methanol	6.19	2.74	5.71	25.28	0.36	0.27	0.08	0.37
U. dioica	ro	P	methanol	7.75	2.93	4.19	28.74	0.44	0.29	0.06	0.42
U. dioica	ro	P	methanol	6.74	2.60	3.31	26.85	0.39	0.25	0.05	0.39
U. dioica	le	T	methanol	29.46	49.07	47.37	89.74	0.83	1.22	0.70	0.91
U. dioica	le	T	methanol	33.45	42.07	46.65	92.93	0.94	1.05	0.69	0.94
U. dioica	le	T	methanol	41.39	47.85	44.36	100.83	1.16	1.19	0.66	1.02
U. dioica	st	T	methanol	8.64	9.57	57.55	31.20	0.24	0.24	0.86	0.32
U. dioica	st	T	methanol	8.08	7.72	50.72	30.47	0.23	0.19	0.75	0.31
U. dioica	st	T	methanol	10.11	7.05	40.06	28.25	0.28	0.18	0.60	0.29
U. dioica	ro	T	methanol	10.42	6.07	6.54	24.28	0.29	0.15	0.10	0.25
U. dioica	ro	T	methanol	9.59	5.94	7.15	22.27	0.27	0.15	0.11	0.23
U. dioica	ro	T	methanol	9.00	5.27	2.09	24.33	0.25	0.13	0.03	0.25
U. dioica	le	L	methanol	26.68	12.91	50.54	74.30	1.00	1.03	0.85	0.77
U. dioica	le	L	methanol	13.53	11.41	50.69	82.02	0.51	0.91	0.85	0.85
U. dioica	le	L	methanol	25.56	11.99	51.02	88.99	0.96	0.96	0.86	0.92
U. dioica	st	L	methanol	7.65	3.75	35.76	28.00	0.29	0.30	0.60	0.29
U. dioica	st	L	methanol	8.11	4.14	37.42	34.55	0.31	0.33	0.63	0.36
U. dioica	st	L	methanol	9.24	3.85	34.26	35.31	0.35	0.31	0.58	0.36
U. dioica	ro	L	methanol	7.18	2.84	1.24	28.70	0.27	0.23	0.02	0.30
U. dioica	ro	L	methanol	13.66	2.72	3.44	32.62	0.51	0.22	0.06	0.34
U. dioica	ro	L	methanol	7.90	2.63	2.52	31.17	0.30	0.21	0.04	0.32
